# Editorial: Chasing brain dynamics at their speed: what can time-varying functional connectivity tell us about brain function?

**DOI:** 10.3389/fnins.2023.1223955

**Published:** 2023-06-13

**Authors:** Maria Rubega, Silvia Francesca Storti, David Pascucci

**Affiliations:** ^1^Section of Rehabilitation, Department of Neuroscience, University of Padua, Padua, Italy; ^2^Department of Engineering for Innovation Medicine, University of Verona, Verona, Italy; ^3^Laboratory of Psychophysics, Ecole Polytechnique Fédérale de Lausanne (EPFL), Lausanne, Switzerland

**Keywords:** brain networks, neural networks, EEG, fMRI, dynamic functional connectivity, dFC

In the past decades, the growing field of network neuroscience has opened new perspectives on the study of the brain and its function. The integration of tools from network analysis and system neuroscience has allowed researchers to explore the properties of brain networks, offering a valuable alternative to traditional methods based on simple subtraction and mass univariate analysis (Sporns, 2010; Behrens and Sporns, 2012). This has led to an exponential growth of connectivity algorithms and methods designed to capture the intrinsic dynamics of human brain networks, both at rest and during active tasks. As a result, a new research direction has emerged. The quantification of spatio-temporal dynamics of functional connectivity (FC) is offering new means to observe a vast repertoire of brain functions.

Despite significant advances in this domain, there are still major challenges to address. This is partly due to the rapid and distributed nature of brain interactions, with large-scale networks that constantly evolve and coordinate activity to produce human perception, cognition, and behavior at sub-second timescales. Additionally, brain network activity can vary widely within and across individuals (Finn et al., 2015; Van De Ville et al., 2021), as well as in clinical conditions and brain disorders (see Miao et al.). Thus, modeling whole-brain network dynamics, accounting for the necessary spatial and temporal resolution at both individual and population levels, remains a crucial goal yet to be fully achieved.

The present Research Topic contains a collection of methodological and empirical studies that touch upon some of the main challenges in the field, collectively providing insight into the current state of research and the potential solutions for advancing the field of dynamic network neuroscience in the future.

## The trade-off between spatial and temporal resolution

Brain network research requires the use of various neurophysiological and neuroimaging approaches, each with unique strengths and weaknesses. The choice of methodology often depends on the research question, portability, and cost, and is typically affected by a trade-off between the spatial and temporal resolution offered by different imaging data types.

Electroencephalography (EEG), due to its high temporal resolution, has the potential to capture non-stationary dynamics in neural activity, including rapid changes that occur on the millisecond scale, such as the timing of individual spikes or synchronization/desynchronization of oscillatory activity across different brain regions. There have been significant developments and enhancements to connectivity analysis using EEG inverse solutions. However, EEG and inverse solutions provide limited spatial resolution compared to functional magnetic resonance imaging (fMRI), restricting the ability to pinpoint neural activity in specific brain regions and interactions between nearby regions. On the one hand, EEG analysis remains suboptimal for approaching research questions that involve subcortical regions or the cerebellar cortex, such as that in Pang et al., where the authors investigated correlations between the language network in temporal lobe epilepsy and the cerebellum. On the other hand, fMRI can provide precise information about the location of neural activity within the brain (including both cortical and subcortical brain areas). Despite the limited temporal resolution of fMRI, which operates at the scale of seconds, remarkable progress has been achieved in developing methods to estimate time-varying connectivity networks or “brain states” from fMRI data (Calhoun et al., 2014; Kringelbach and Deco, 2020; Lurie et al., 2020; Pezzulo et al., 2021). Combining and integrating EEG and fMRI through novel methods shows promise to overcome the limitations of both techniques and benefit from their advantages due to their non-invasive nature (Formaggio et al., 2011; Hinne et al., 2014; Pascucci et al., 2022).

## The curse of dimensionality

The techniques for analyzing, extracting, and interpreting brain networks have evolved rapidly, progressing from simple temporal correlation measures between multiple regions to more complex linear and non-linear models that account for time-varying and causal effects. FC is typically computed using bivariate correlations and autoregressive models and is useful for both exploratory and confirmatory research. Recent developments in FC have enabled the estimation of time-frequency matrices that show direct and directed interactions between brain networks (Chang and Glover, 2010; Sakoǧlu et al., 2010; Zhang et al.). This type of connectivity is known as “time-varying” or “dynamic” functional connectivity (dFC) and has been developed primarily, but not only, in the domain of EEG source imaging (Milde et al., 2010; Storti et al., 2017; Rubega et al., 2019; Pascucci et al., 2020).

**Figure 1:**
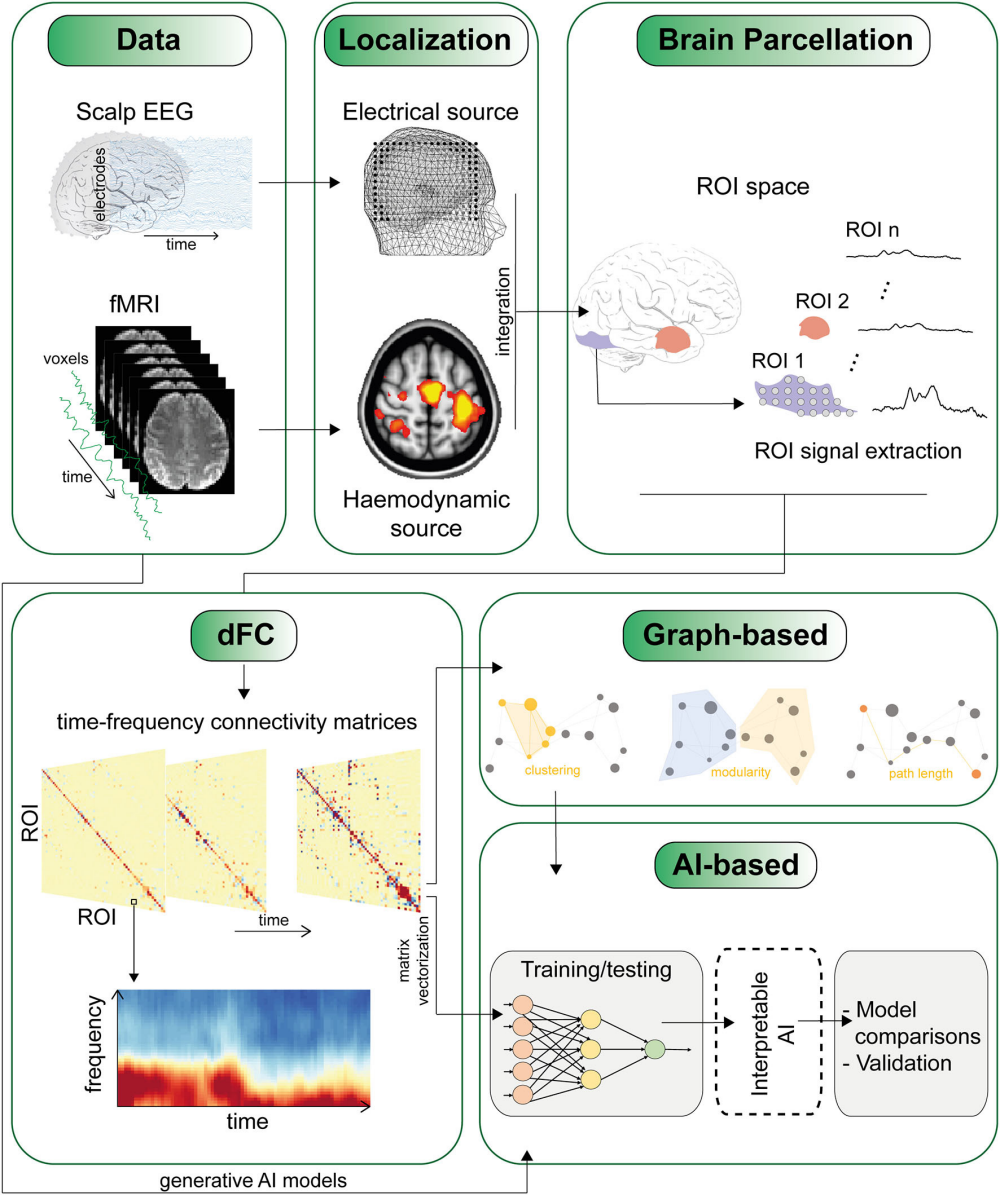
The figure depicts one of several potential pipelines for studying dynamic functional connectivity (dFC). The sequence of panels highlights some of the major challenges, starting from the integration of data from different modalities (such as EEG and fMRI) to the definition of regions of interest (ROI) and the derivation of time-varying connectivity matrices. An essential final step involves techniques to condense and extract meaningful insights from high-dimensional connectivity matrices. This can involve employing network analysis using traditional graph metrics, as well as by leveraging AI methods that utilize cross-validation techniques and generative AI models (refer to the main text).

However, as the level of detail increases, such as with time-frequency matrices, the dimensionality of the data rises, sometimes with matrices up to four dimensions, making analysis and interpretation more challenging (see Figure 1). Therefore, a new research avenue is focusing on developing methods that can compress and reduce the dimensionality of FC matrices to extract latent patterns and represent the results in a more tractable, lower dimensional space, as shown in the study by Jia et al..

The challenge of modeling brain networks is further compounded by the complex and highly non-linear nature of neuronal interactions, which cannot always be accurately captured by models assuming linear relationships such as correlation and autoregressive models (Stephan et al., 2008). A potential solution is the use of more advanced machine learning methods. However, even deep convolutional neural networks, which are among the traditional “gold standard” in deep learning, may not be optimal for analyzing complex brain networks in EEG or fMRI data, due to the inherent noise, sparsity and non-stationarity of imaging data. Additionally, some machine learning methods, including deep learning models, are often considered “black boxes” because they do not provide any explanations or rationales for their outcomes. Therefore, alternative approaches suggest moving toward explainable artificial intelligence (XAI) methods to ensure that models are transparent and understandable to end-users. To address these issues, Lin et al. proposed a convolutional recurrent neural network (CRNN) that can extract high-level features and preserve sequential information in dFC networks. Alternatively, graph neural networks (GNNs) offer a more flexible and realistic approach, allowing for insights into FC between different regions of interest. In particular, EEG- or fMRI-GNN can visualize and learn connectivity between important nodes, addressing the issue of interpretability. The comparison between CNN-based and GNN-based methods is a relevant topic, but aspects such as the choice of appropriate FC measures to define the brain graph still need to be explored. A further advancement of this approach involves using graph-generating networks (GGNs) instead of pre-defined graphs, as proposed by Lin et al. for epilepsy research. Hou et al. (2022) have also contributed to this area. Importantly, a key aspect and potential limitation of using deep learning methods is that they require a large amount of (unsupervised) data for training to achieve good performance. This can be challenging given the variety of tasks and small sample sizes typically used in imaging studies.

## Future directions

Brain network research foreshadows the possibility of gaining a rich and physiologically grounded understanding of brain function at the level of distributed and dynamic networks from which it emerges. As a result, this field has attracted interest and contributions from a variety of disciplines, serving as one of the most compelling examples of the integration of imaging and analysis techniques in contemporary neuroscience. However, this has also highlighted the need to combine established and emerging tools to improve the spatial and temporal resolution of brain data, as well as to develop data reduction techniques. We anticipate these to be at the forefront of research in the coming decades.

## Author contributions

All authors listed have made a substantial, direct, and intellectual contribution to the work and approved it for publication.
